# An Integrated Multi-Omics and Artificial Intelligence Framework for Advance Plant Phenotyping in Horticulture

**DOI:** 10.3390/biology12101298

**Published:** 2023-09-30

**Authors:** Danuta Cembrowska-Lech, Adrianna Krzemińska, Tymoteusz Miller, Anna Nowakowska, Cezary Adamski, Martyna Radaczyńska, Grzegorz Mikiciuk, Małgorzata Mikiciuk

**Affiliations:** 1 Department of Physiology and Biochemistry, Institute of Biology, University of Szczecin, Felczaka 3c, 71-412 Szczecin, Poland; anna.nowakowska@usz.edu.pl; 2 Polish Society of Bioinformatics and Data Science BIODATA, Popiełuszki 4c, 71-214 Szczecin, Poland; adrianna.krzeminska95@gmail.com (A.K.); tymoteusz.miller@usz.edu.pl (T.M.); 3 Institute of Biology, University of Szczecin, Wąska 13, 71-415 Szczecin, Poland; 221392@stud.usz.edu.pl; 4 Institute of Marine and Environmental Sciences, University of Szczecin, Wąska 13, 71-415 Szczecin, Poland; 5 Sanprobi Sp. z o. o. Sp. k., Kurza Stopka 5/C, 70-535 Szczecin, Poland; martyna.radaczynska@sanprobi.pl; 6 Department of Horticulture, Faculty of Environmental Management and Agriculture, West Pomeranian University of Technology in Szczecin, Słowackiego 17, 71-434 Szczecin, Poland; grzegorz.mikiciuk@zut.edu.pl; 7 Department of Bioengineering, Faculty of Environmental Management and Agriculture, West Pomeranian University of Technology in Szczecin, Słowackiego 17, 71-434 Szczecin, Poland; malgorzata.mikiciuk@zut.edu.pl

**Keywords:** multi-omics, artificial intelligence, machine learning, plant phenotyping, data integration, precision horticulture

## Abstract

**Simple Summary:**

The future of plant biology, particularly rapidly advancing precision horticulture and predictive breeding, will require the transformation of huge volumes of multi-omics data into structured information and valuable knowledge, representing a key challenge. This review aims to delve into the transformative potential of integrating multi-omics data and artificial intelligence (AI) for a more comprehensive, high-throughput approach to plant phenotyping in horticultural research. We argue that the union of these advanced techniques can provide a robust analytical framework that can handle the complexity of plant biology, thus surmounting the limitations of traditional phenotyping methods. Our discussion also acknowledges the technical and non-technical challenges associated with this integration, critically evaluating their advantages and limitations, proposing potential solutions, and outlining promising future prospects.

**Abstract:**

This review discusses the transformative potential of integrating multi-omics data and artificial intelligence (AI) in advancing horticultural research, specifically plant phenotyping. The traditional methods of plant phenotyping, while valuable, are limited in their ability to capture the complexity of plant biology. The advent of (meta-)genomics, (meta-)transcriptomics, proteomics, and metabolomics has provided an opportunity for a more comprehensive analysis. AI and machine learning (ML) techniques can effectively handle the complexity and volume of multi-omics data, providing meaningful interpretations and predictions. Reflecting the multidisciplinary nature of this area of research, in this review, readers will find a collection of state-of-the-art solutions that are key to the integration of multi-omics data and AI for phenotyping experiments in horticulture, including experimental design considerations with several technical and non-technical challenges, which are discussed along with potential solutions. The future prospects of this integration include precision horticulture, predictive breeding, improved disease and stress response management, sustainable crop management, and exploration of plant biodiversity. The integration of multi-omics and AI holds immense promise for revolutionizing horticultural research and applications, heralding a new era in plant phenotyping.

## 1. Introduction

### 1.1. Background of Horticulture and Plant Phenotyping

Horticulture, one of the most integral sectors within the broader sphere of agriculture, has played a pivotal role in human civilization. It has facilitated our transition from nomadic hunter–gatherers to settled agricultural societies. As a field, horticulture encompasses the science, technology, and art involved in the cultivation, propagation, processing, and marketing of ornamental plants, flowers, fruits, vegetables, nuts, seeds, and herbs [1,2]. At the core of horticulture lies the concept of plant phenotyping, the comprehensive assessment of complex plant traits such as growth, development, tolerance, resistance, architecture, physiology, ecology, and yield quality and quantity under a range of environmental conditions. The intricate relationship between a plant’s phenotype and its environment is modulated by its genotype, forming the basis for plant phenomics [3,4,5].

Over the years, plant phenotyping has been paramount in assessing plant characteristics, enabling the development of improved crop varieties, and paving the way for increased agricultural and horticultural productivity and resilience. However, the traditional methods of plant phenotyping, often manual, time-consuming, and subject to human error, have been unable to keep pace with the rapid advancements in high-throughput genotyping technologies [6,7]. The demand for food is expected to grow substantially in the next decades. To meet the challenges of this global growth in a context of climate change, a better understanding of genotype–phenotype relationships is crucial to improve production capacities. Plant research is witnessing an unprecedented revolution in the acquisition of various data such as phenotypic and multi-omic data, which generates terabytes of data associated with the results of large-scale phenotypic experiments carried out in environments with different conditions. The disparity between genotyping and phenotyping capabilities has become a critical bottleneck in our quest to ensure global food security and sustainable agriculture. As such, the need for innovative and advanced plant phenotyping techniques has never been more pressing [8,9].

To address these challenges, we stand at the brink of integrating cutting-edge technologies such as multi-omics approaches and artificial intelligence into horticulture. By leveraging these technologies, we seek to establish a more holistic and nuanced understanding of plant biology. This, in turn, promises unprecedented insights into plant phenotypes and the ability to breed more resilient and productive crops [10,11,12].

In the following sections, we will delve into the significance of multi-omics and AI in the contemporary horticulture landscape and propose an integrated framework that harnesses these technologies for advanced plant phenotyping.

### 1.2. Need for Advanced Techniques in Plant Phenotyping

The last few decades have witnessed a significant shift in the realm of plant phenotyping, primarily driven by the advent of advanced high-throughput genotyping technologies. These technologies have enabled the generation of vast genomic datasets, prompting a newfound appreciation for the genetic complexity underpinning plant phenotypes [13,14]. However, this rapid proliferation of genotypic data has not been matched by comparable strides in phenotypic data acquisition, leading to a notable phenotyping bottleneck. This disparity has underscored the need for more advanced and high-throughput plant phenotyping techniques [15,16].

**Figure 1:**
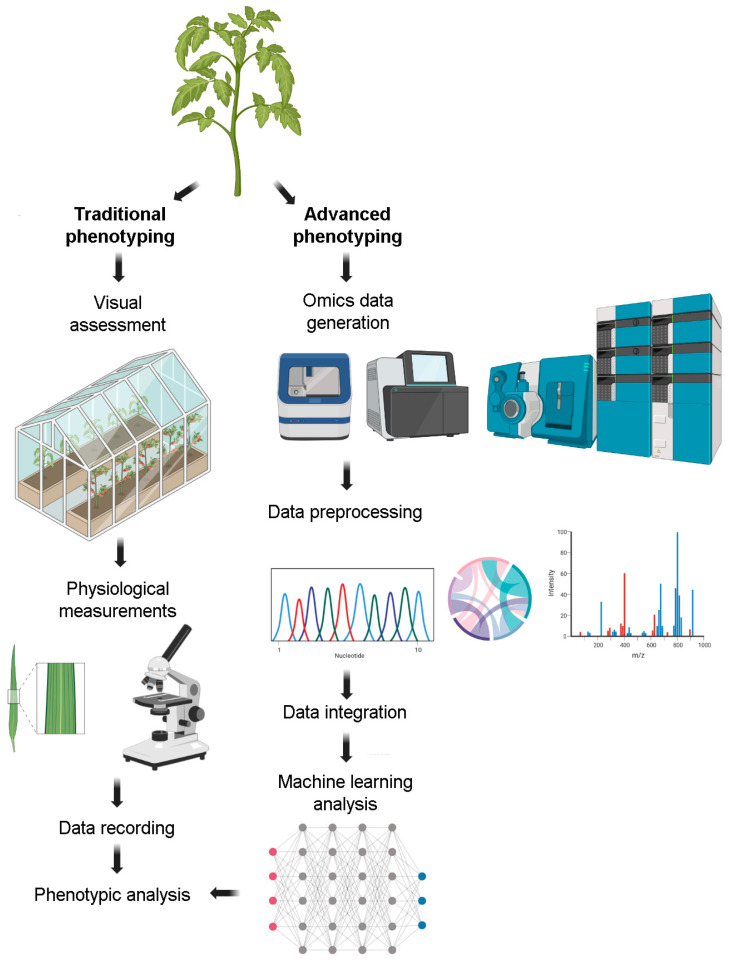
Overview of traditional and advanced phenotyping techniques.

Traditional phenotyping methods are often labor-intensive, subjective, and suffer from low throughput, making it challenging to capture the dynamic nature of plant traits across different growth stages and environmental conditions (Figure 1 and Table 1). Furthermore, these methods generally focus on observable traits, overlooking subcellular processes and interactions that contribute significantly to the overall plant phenotype [6,7,17]. Consequently, it has become evident that the next frontier in plant phenotyping necessitates a paradigm shift towards more precise, objective, and high-throughput methodologies. This shift should be equipped to capture the complexity and dynamics of plant phenotypes at different scales, from cellular processes to whole-plant traits, and under varying environmental conditions [18,19,20].

Advanced techniques such as imaging technologies, sensor-based measurements, and high-throughput screening platforms are increasingly being incorporated into plant phenotyping, paving the way towards more efficient and precise data collection (Figure 1 and Table 1). However, these techniques invariably generate vast and complex datasets, necessitating robust data analysis strategies [21,22,23]. Multi-omics methodologies promise a holistic view of the plant system by integrating genomic, transcriptomic, proteomic, and metabolomic data, among others. Meanwhile, artificial intelligence and machine learning offer powerful tools for deciphering complex patterns within these large datasets, enabling more insightful and predictive models of plant phenotypes [24,25,26]. Thus, the integration of these advanced techniques within plant phenotyping not only holds the potential to break the phenotyping bottleneck, but also promises to usher in a new era of precision horticulture.

**Table 1:** Comparison of traditional and advanced phenotyping methods.

Method	Description	Advantages	Limitations
Traditional Phenotyping	Traditional phenotyping in horticulture primarily relies on visual assessment and manual measurements of plant traits, such as plant height, flower color, fruit size, and disease symptoms [5].	1. Simple and cost-effective [6].	1. Time-consuming and labor-intensive [7].
		2. Easy to conduct without requiring specialized training or tools [7].	2. Limited in scope and depth, typically only capturing superficial traits [6].
			3. Subjective, with potential for inconsistency and error [5].
Advanced Phenotyping (Multi-Omics)	Advanced phenotyping involves comprehensive molecular profiling of the plant, using techniques such as genomics, transcriptomics, proteomics, and metabolomics [26].	1. Provides in-depth understanding of plant biology at the molecular level [25].	1. Requires specialized equipment and training [27].
		2. Can reveal information about complex traits and processes [27].	2. Data analysis can be complex, given the volume and complexity of multi-omics data [25].
Advanced Phenotyping (AI/ML)	AI/ML-based phenotyping involves the use of machine learning algorithms to analyze and interpret complex plant data, such as images, spectral data, or multi-omics data [10].	1. Can handle large volumes of complex data [28].	1. Requires substantial computational resources and expertise [28].
		2. Provides objective and consistent analyses [29,30].	2. Model selection and interpretation can be challenging [29].
		3. Can uncover patterns and relationships that are not evident to humans [30].	3. ‘Black box’ nature of some ML algorithms can lead to transparency and trust issues [30].

### 1.3. Brief Overview of Multi-Omics and AI Techniques

Multi-omics and artificial intelligence (AI) represent two technological advancements that hold significant potential to revolutionize horticulture and plant phenotyping. These approaches, when combined, have the potential to offer unprecedented insights into the complexities of plant systems and enable the development of highly accurate and predictive models of plant phenotypes [27,28,29].

The term ‘multi-omics’ refers to the integrative study of various ‘omic’ disciplines, which individually focus on a particular biological system. These include genomics, transcriptomics, proteomics, metabolomics, and others [30,31]. Each of these omics layers offers a unique perspective on the functional components of a biological system. However, by considering these layers separately, the holistic picture of how these components interact and contribute to the overall phenotype is lost. This is why multi-omics represents an advance [17,32]. Multi-omics approaches aim to integrate data from various omics layers to uncover the complex interactions and regulatory mechanisms that underlie the observable characteristics or phenotypes of an organism. In the context of plant phenotyping, multi-omics can provide comprehensive insights into the dynamic interplay between genetic makeup, environmental influence, and plant phenotype [33,34].

Artificial intelligence (AI) refers to the simulation of human intelligence processes by machines, especially computer systems. This involves learning (acquiring information and rules), reasoning (using rules to reach conclusions), and self-correction. Machine learning (ML), a subset of AI, involves the development of algorithms that allow computers to learn from and make decisions based on data [35,36].

In horticultural research, AI and ML techniques hold the potential to transform the analysis of large and complex multi-omics datasets. They can uncover hidden patterns within the data, generate new hypotheses, and predict future outcomes with high accuracy. Techniques such as deep learning, a subfield of ML that imitates the functioning of the human brain in processing data, are being increasingly employed to decipher the complex patterns within multi-omics data [37,38,39]. The application of AI and ML in plant phenotyping can facilitate the identification of key features associated with important traits, thus aiding in the development of improved plant varieties. When coupled with multi-omics data, these techniques provide a powerful tool to comprehensively understand and accurately predict plant phenotypes [40,41].

In summary, the combination of multi-omics approaches and AI techniques represents a promising pathway to address the current limitations in plant phenotyping and heralds a new era in horticultural research.

## 2. Advancements in Plant Phenotyping

### 2.1. Traditional Methods of Plant Phenotyping in Horticulture

Plant phenotyping has always been a cornerstone of horticultural research and breeding programs. Traditionally, phenotypic data were obtained through manual measurements and visual inspections, techniques that are rooted in centuries of agricultural practice [42].

These traditional phenotyping methods encompass a broad array of approaches, each of which focuses on a specific plant characteristic or trait. Here, we outline some of the most prevalent traditional phenotyping techniques:

- Visual inspection: This is perhaps the most common and straightforward method of plant phenotyping. Researchers visually inspect plants for specific traits, such as color, shape, size, and disease symptoms. This method is cost-effective and straightforward but is also highly subjective and can lead to inconsistencies due to variability in human judgment [43];
- Manual measurements: A host of plant traits, such as plant height, leaf area, and fruit size are often measured manually using instruments such as rulers, calipers, or leaf area meters. While this method is more objective than visual inspection, it is time-consuming, labor-intensive, and may cause physical damage to the plant, thereby limiting its applicability for large-scale studies [6];
- Destructive sampling: Certain plant traits, particularly those related to plant physiology or internal structures, necessitate destructive sampling. This involves harvesting parts or whole plants to carry out measurements. Examples include determining the dry weight, nutrient content, or internal fruit quality. Although this method can provide highly accurate measurements, it is not suitable for longitudinal studies as it prevents the further assessment of the same plant [4];
- Greenhouse and field trials: For assessing plant performance under different environmental conditions or treatments, greenhouse or field trials are often conducted. These trials involve growing plants under controlled or real-world conditions, respectively, and recording various phenotypic traits. Although valuable for assessing real-world plant performance, these trials can be resource-intensive and subject to environmental variability [44].

Despite their extensive usage, traditional plant phenotyping methods have several limitations, particularly in the context of large-scale studies and high-throughput screening. These constraints have necessitated the development of more advanced, efficient, and high-throughput phenotyping techniques, which are discussed in the next section.

### 2.2. Limitations of Traditional Methods

While traditional methods have played an indispensable role in our understanding of plant phenotypes, they are not without their limitations. As we move towards an era of large-scale genomics and high-throughput screening, these limitations are becoming increasingly apparent:

- Labor-intensive and time-consuming: One of the most significant drawbacks of traditional plant phenotyping methods is that they are often manual and therefore labor-intensive and time-consuming. This makes them unsuitable for large-scale studies where thousands of plants may need to be phenotyped [45];
- Subjectivity and inconsistencies: Methods such as visual inspection are subjective and can result in significant inconsistencies due to variability in human judgment. Furthermore, manual measurements are prone to errors, which can compromise the accuracy of the phenotypic data [46];
- Low throughput: Traditional phenotyping methods generally have a low throughput, meaning that they can only phenotype a limited number of plants within a given timeframe. This is a significant constraint in modern horticultural research and breeding programs where large plant populations often need to be phenotyped [6,17];
- Destructive nature: Some traditional phenotyping methods, such as destructive sampling, prevent the further assessment of the same plant and are therefore not suitable for longitudinal studies where the same plant needs to be assessed at different time points [4];
- Inability to capture subcellular processes: Traditional methods generally focus on observable traits and are unable to capture subcellular processes and interactions that significantly contribute to the overall plant phenotype [4,46];
- Environmental variability: Greenhouse and field trials are subject to environmental variability, which can introduce a significant amount of noise into the phenotypic data and complicate the interpretation of the results [43,47].

As such, there is an increasing recognition within the horticultural community of the need to overcome these limitations through the application of more advanced and high-throughput plant phenotyping techniques. These techniques, combined with the power of multi-omics and AI, have the potential to revolutionize our understanding of plant phenotypes and facilitate the development of more resilient and productive crop varieties.

### 2.3. Advancements and Their Potential

The past decade has witnessed a surge in innovative plant phenotyping techniques that promise to address the limitations of traditional methods. These advancements leverage cutting-edge technologies to enable more efficient, precise, and high-throughput phenotyping. Here, we outline some of these techniques and their potential impact on horticultural research:

- High-throughput phenotyping platforms: High-throughput phenotyping (HTP) platforms, both in the greenhouse and field, employ automated systems to non-invasively measure multiple plant traits simultaneously. These platforms utilize a combination of imaging technologies, sensor-based measurements, and robotics to phenotype large plant populations in a relatively short time. HTP platforms significantly reduce manual labor and improve the objectivity and consistency of phenotypic measurements [48,49].
- Imaging technologies: Innovations in imaging technologies have revolutionized plant phenotyping. These technologies provide non-invasive, objective, and high-resolution measurements of a wide range of plant traits. Techniques such as RGB imaging, hyperspectral imaging, thermal imaging, 3D imaging, and fluorescence imaging can capture various aspects of plant physiology, morphology, and health. For example, RGB imaging can be used to assess plant color and size, while hyperspectral imaging can provide insights into plant nutrient status and disease resistance [50,51].
- Sensor-based measurements: The advent of various sensor technologies has facilitated the capture of precise and continuous phenotypic data. These include sensors for measuring soil moisture, leaf temperature, light intensity, and plant water status, among others. Sensor-based measurements provide real-time insights into plant responses to environmental changes, allowing for more nuanced understanding of plant–environment interactions [52,53].
- Drones and remote sensing: Drones equipped with advanced imaging systems and sensors provide a powerful tool for large-scale field phenotyping. They can capture high-resolution, multi-dimensional images of entire fields, enabling the assessment of spatial variability in plant traits across large areas. Similarly, remote sensing technologies allow for large-scale monitoring of crop health, yield, and environmental conditions [54,55].
- Integration of multi-omics and AI: The integration of multi-omics approaches with advanced phenotyping techniques can provide a holistic view of the plant system, uncovering the complex interactions and regulatory mechanisms that underlie observable plant traits. Moreover, AI and machine learning techniques can be leveraged to analyze the large and complex datasets generated by these methods, revealing hidden patterns and predictive models of plant phenotypes [56,57,58]. These advancements have the potential to revolutionize plant phenotyping, breaking the existing bottleneck and paving the way for more insightful and predictive horticultural research. Through these advancements, we can expect to see significant strides in our understanding of plant biology and the development of more productive and resilient crop varieties.

## 3. Introduction to Multi-Omics

### 3.1. Overview of Genomics, Transcriptomics, Proteomics, and Metabolomics

The ‘omics’ disciplines represent a comprehensive approach to studying various biological systems in a holistic and integrative manner. These disciplines, when combined under the umbrella of ‘multi-omics’, allow us to understand the complex interplay between different layers of biological information. Here, we provide an overview of the key omics disciplines: genomics, transcriptomics, proteomics, and metabolomics (Table 2).

Genomics refers to the study of an organism’s entire genome or the complete set of DNA, including all its genes. It involves understanding the structure, function, evolution, and mapping of genomes. Genomics allows researchers to study complex genetic traits and understand how multiple genes can influence these traits. In the context of plant phenotyping, genomics can provide insights into the genetic determinants of various plant traits and aid in the development of marker-assisted selection strategies [59,60]. Whole-genome sequencing (WGS) provides an in-depth, comprehensive view of the plant genome, and can help discover novel genes and regulatory elements that were previously uncharacterized. Genotyping-by-sequencing, on the other hand, is a cost-effective method for identifying single nucleotide polymorphisms (SNPs) and small insertions and deletions (INDELs) [61,62,63]. This method is particularly valuable for genetic mapping, marker-assisted breeding, and population genetic studies. SNPs and other genetic variations are the basis of genetic diversity and can influence various traits of interest in horticulture, such as fruit size, color, flavor, and resistance to diseases or pests. For example, an SNP in a particular gene may cause a change in a protein’s function, leading to a change in a plant’s phenotype [64,65,66]. Overall, genomics, powered by NGS technologies, has opened up vast opportunities for understanding the complex genetic architecture of plants and accelerating genetic improvement in horticulture [67,68,69].

Transcriptomics (RNA-seq) involves the study of the transcriptome, the complete set of RNA transcripts produced by the genome under specific circumstances or in a specific cell. Transcriptomics provides insights into gene expression patterns, allowing researchers to understand which genes are turned ‘on’ or ‘off’ during different developmental stages or under different environmental conditions. This information can reveal how genetic information is translated into functional outcomes, and it can also help identify genes that play a critical role in specific plant traits [32,60,70]. For example, in horticultural research, RNA-seq could be used to understand the transcriptomic changes that occur during fruit ripening or in response to disease [71,72,73]. The high-resolution data generated by transcriptomics not only provides a snapshot of gene activity at a specific moment, but can also be used to understand the dynamic nature of gene expression. Such understanding can lead to the identification of key molecular mechanisms and regulatory networks in plants, which can significantly influence horticultural practices and crop improvement strategies. Overall, transcriptomics serves as an essential bridge between the genome and the phenotype, contributing significantly to the elucidation of the functional elements of the genome and their roles in horticulture [74,75,76]. 

Proteomics studies the entire set of proteins expressed by a genome, which includes their interactions, modifications, localization, and functions. By studying the structures, functions, and interactions of proteins, proteomics can provide valuable insights into the cellular mechanisms underlying various plant traits. Proteomic analyses can also reveal post-translational modifications, protein–protein interactions, and the impact of environmental factors on protein function [32,34]. This ‘omics’ approach complements genomics and transcriptomics, providing a more direct link to cellular function and phenotype since proteins are the primary effectors of cellular processes. In horticulture, proteomics can be used to identify key proteins involved in essential biological processes such as photosynthesis, respiration, signaling pathways, and stress responses. For instance, a comparative proteomic analysis between disease-resistant and susceptible plant varieties could reveal proteins that contribute to disease resistance. Furthermore, protein–protein interaction studies can shed light on the complex protein networks that regulate plant development and responses to environmental cues. For example, understanding the protein interactions involved in the fruit ripening process could help in the development of strategies to enhance fruit quality and shelf life [77,78,79]. 

Metabolomics involves the systematic study of the unique chemical fingerprints that specific cellular processes leave behind, i.e., the study of their small-molecule metabolite profiles. Metabolomics can provide information about the physiological status of a plant and its response to environmental conditions. By comparing the metabolomes of different plants or the same plant under different conditions, researchers can identify changes in metabolic pathways that may influence specific plant traits [32,80,81].

**Table 2:** Overview of multi-omics techniques.

Type of ‘Omics’	Definition	Common Methods Used	Applications in Horticulture
Genomics	The study of the complete set of genes (the genome) in a species and their functions [63].	Whole-genome sequencing (WGS), genotyping by sequencing (GBS) [68].	Pangenome analysis, plant breeding, genetic diversity analysis, disease resistance research [69].
Transcriptomics	The study of the complete set of RNA transcripts produced by the genome under specific circumstances [70].	RNA sequencing (RNA-seq), single-cell RNA sequencing (scRNA-seq), microarray analysis [71].	Understanding plant response to stress, gene expression studies, identification of key regulatory genes [72].
Proteomics	The study of the complete set of proteins as expressions of genes and their functions [73].	Two-dimensional gel electrophoresis, mass spectrometry [74].	Studying protein interaction networks, protein expression analysis, discovering disease resistance proteins [75].
Metabolomics	The study of the complete set of small-molecule chemicals found within a biological sample [76].	Gas chromatography–mass spectrometry (GC–MS), liquid chromatography–mass spectrometry (LC–MS) [77].	Profiling of plant-targeted and untargeted metabolites, understanding plant metabolic pathways, flavor and fragrance research [72].

Together, these omics disciplines provide a comprehensive view of the biological system, from the genetic blueprint (genome) to its functional molecules (transcriptome, proteome, and metabolome). The integration of these layers using a multi-omics approach can reveal the complex networks and interactions that shape the observable characteristics of a plant, thus providing a more holistic understanding of plant phenotypes at the molecular level. Furthermore, it can help in identifying the molecular markers associated with desirable traits, which can be used for plant breeding and genetic improvement in horticulture.

### 3.2. Significance of Integrating Multi-Omics Data in Horticulture

The integration of multi-omics data is a powerful approach that can enhance our understanding of plant biology and significantly accelerate progress in horticultural research and breeding programs. Here, we outline the significance of integrating multi-omics data in horticulture:

- Comprehensive view of biological systems: A primary advantage of multi-omics integration is the comprehensive and holistic perspective it provides of biological systems. By combining genomics, transcriptomics, proteomics, and metabolomics, researchers can explore multiple layers of biological information simultaneously. This approach can reveal how genetic variants influence gene expression, protein production, and metabolite levels, and consequently, observable plant traits [82,83,84].
- Uncovers complex interactions and regulatory mechanisms: Integration of multi-omics data can uncover the complex interactions and regulatory mechanisms that underlie plant phenotypes. For instance, by correlating genomic data with transcriptomic, proteomic, or metabolomic data, researchers can identify how changes in the DNA sequence impact gene expression, protein production, and metabolite levels. This information can illuminate the mechanisms through which genetic variations contribute to observable traits [84,85,86].
- Enhances predictive power: The integration of multi-omics data can enhance the predictive power of models used to forecast plant traits. By incorporating data from multiple omics layers, these models can account for the interplay between different biological processes, leading to more accurate predictions [85,87].
- Facilitates precision breeding: Multi-omics integration can facilitate precision breeding by identifying molecular markers associated with desirable plant traits across multiple biological layers. This allows breeders to select for these traits with greater precision, leading to the development of improved plant varieties [88,89].
- Improves understanding of plant–environment interactions: Through the integration of multi-omics data, researchers can gain a deeper understanding of how plants interact with their environment. This can reveal how various environmental factors influence gene expression, protein production, and metabolic pathways, thereby affecting plant growth, development, and response to stress [90,91].
- Aids in disease diagnosis and management: By providing a comprehensive view of plant biology, multi-omics integration can aid in the diagnosis and management of plant diseases. For example, it can help identify molecular markers associated with disease resistance, guide the development of disease-resistant plant varieties, and inform disease management strategies [78,92].

### 3.3. Exploring Specific Molecular Pathways in Horticulture

The application of multi-omics and AI technologies in horticulture enables in-depth exploration and understanding of complex molecular pathways integral to plant growth, disease resistance, and stress responses.

#### 3.3.1. Plant Growth and Development

Plant growth and development are orchestrated by a complex network of genes and their interactions. Through multi-omics techniques, we can gain a deeper understanding of these molecular mechanisms and the key players involved:

- Genomic insights: Genomics offers a comprehensive view of a plant’s genetic makeup, shedding light on crucial genes involved in growth and development. For instance, genes in the auxin signaling pathway, a critical regulator of plant cell elongation and organ shape, can be identified and their sequences analyzed. Genomic variations such as single nucleotide polymorphisms (SNPs) or insertions and deletions (INDELs) within these genes can be linked to phenotypic variations, contributing to our understanding of plant morphology and development [93,94,95].
- Transcriptomic profiling: Transcriptomics takes this a step further by studying the expression patterns of these genes. It can provide insights into when and where specific genes are turned on or off during a plant’s life cycle, adding another layer of complexity to our understanding of plant development. For example, RNA-seq technology can be used to monitor gene expression changes in the auxin pathway throughout different developmental stages or in response to external stimuli [96,97,98].
- Metabolomic analysis: Metabolomics complements these genetic and transcriptional studies by investigating the metabolic changes that accompany plant growth and development. It can identify and quantify the multitude of metabolites in a plant, revealing the biochemical pathways that are active at various stages of development. For instance, metabolomic studies can show how the auxin hormone and other related metabolites fluctuate during plant development, providing more tangible measures of plant physiological changes [99,100,101].
- Role of AI and ML: The integration and analysis of this high-dimensional multi-omics data can be challenging. This is where AI and ML come into play. Advanced AI and ML techniques can be used to recognize patterns within this complex data, facilitating the prediction of gene function or plant phenotypic traits. For instance, AI algorithms could predict how changes in the expression of genes in the auxin pathway could impact plant growth or morphology, which could then be experimentally validated [102,103,104].

#### 3.3.2. Disease Resistance Pathways

Plants have evolved a variety of disease resistance pathways to protect themselves against a diverse range of pathogens. These pathways are complex and involve many different genes, proteins, and metabolites. Multi-omics approaches provide an invaluable toolset for understanding these processes on a molecular level:

- Genomic studies: One of the key components in disease resistance pathways is resistance (R) genes. Genomics allows us to analyze genetic variations, such as SNPs and INDELs, within these R genes, which can provide information about a plant’s potential to resist specific diseases [105,106,107];
- Transcriptomic analysis: To understand when and how R genes function in response to pathogen attacks, transcriptomics can be employed. For example, RNA-seq analysis can be used to monitor R gene expression levels upon exposure to different pathogens. This allows us to observe the activation of the disease resistance pathways and to identify the pathogens against which these pathways are effective [108,109,110];
- Proteomic insights: Proteomics can help in understanding the post-transcriptional and post-translational modifications that R proteins undergo during pathogen attacks. These modifications can influence the function and activity of R proteins. By identifying the modified proteins and their modifications, proteomics can provide insights into the mechanisms by which R proteins confer disease resistance [111,112,113];
- Metabolomic studies: Plants respond to pathogen attacks by producing various metabolites that help combat the invaders. Metabolomics can identify and quantify these defensive metabolites, such as phytoalexins, which are synthesized in response to microbial infection. Metabolomic profiles can provide a snapshot of a plant’s metabolic state under pathogen attack, contributing to our understanding of the biochemical aspects of plant defense mechanisms [114,115,116];
- AI/ML in disease resistance studies: Each ‘omics’ layer adds a piece to the puzzle of plant disease resistance. However, integrating and interpreting this vast and complex multi-omics data can be challenging. AI/ML models offer powerful tools to decipher these complexities, enabling the prediction of disease resistance based on multi-omics profiles. For instance, ML algorithms can be trained on genomic, transcriptomic, proteomic, and metabolomic data to predict a plant’s resistance to a specific disease. These predictions can be tested and validated experimentally, allowing for the continuous refinement and improvement of the models [117,118,119].

#### 3.3.3. Stress Response Pathways

Plants, as sessile organisms, are exposed to a myriad of environmental stresses, including drought, salinity, and extreme temperatures. Understanding how plants respond to these stresses at a molecular level is crucial for improving crop resilience. Multi-omics approaches provide a comprehensive toolset for unraveling these complex stress response pathways:

- Genomic studies: Genomics offers the ability to identify genes implicated in stress responses. For instance, several drought, salinity, and temperature-responsive genes have been identified in various plant species. These genes often include those encoding transcription factors, which play a pivotal role in regulating the expression of other stress-responsive genes. Analyzing the sequence and structural variations within these genes can help predict a plant’s potential to withstand different environmental stresses [120,121,122];
- Transcriptomic analysis: Transcriptomic studies can track the expression of stress-responsive genes during exposure to different stress conditions. For instance, RNA-seq analysis can reveal up-regulation or down-regulation of specific genes in response to drought, salinity, or temperature stress. This provides a dynamic view of how a plant’s transcriptome changes in response to environmental stressors [123,124,125];
- Proteomic insights: Proteomics complements these genomic and transcriptomic studies by providing insights into stress-responsive proteins. For instance, certain proteins might be upregulated during stress conditions to protect plant cells from damage. Proteomics can identify these proteins and monitor their abundance during different stress conditions, thereby providing insights into a plant’s proteomic response to stress [68,126,127];
- Metabolomic studies: Metabolomics adds another layer of understanding by investigating the metabolic changes under stress conditions. Certain metabolites may accumulate in response to stress as part of a plant’s defense mechanism. These could include osmolytes for drought and salinity stress or heat-shock proteins for thermal stress. Metabolomic profiling can reveal these stress-induced metabolic changes, providing a holistic view of a plant’s biochemical response to stress [101,128,129];
- Role of AI/ML in studying stress responses: The integration of multi-omics data gives a comprehensive picture of a plant’s response to stress. However, this data is high-dimensional and complex, presenting a challenge for traditional data analysis methods. AI/ML techniques offer robust tools for managing this complexity. They can identify key molecular players in stress responses by detecting patterns across the multi-omics datasets. Furthermore, AI/ML models can be trained to predict a plant’s stress response based on its multi-omics profile [119,130,131];

Through the integration of multi-omics data and AI/ML analyses, we can achieve a deeper understanding of plant stress responses. This knowledge is vital for breeding more resilient crops and for developing more effective strategies for stress management in horticulture.

## 4. Introduction to Artificial Intelligence and Machine Learning

### 4.1. Overview of AI and Machine Learning

Artificial intelligence (AI) and machine learning (ML) are interdisciplinary fields of computer science that have seen tremendous growth and interest in recent years, offering a myriad of applications across various domains, including horticulture. AI refers to the simulation of human intelligence processes by machines, especially computer systems. These processes include learning (the acquisition of information and rules for using the information), reasoning (using the rules to reach approximate or definite conclusions), and self-correction. AI is a broad field that encompasses many subdomains, one of which is machine learning [132,133]. AI systems can be categorized into two types:

- Narrow AI, which is designed to perform a narrow task (e.g., facial recognition or internet searches) and is what we currently have, and
- General AI, which refers to systems that possess the ability to perform any intellectual task that a human being can do; this are still a largely theoretical concept [134,135].

**Figure 2:**
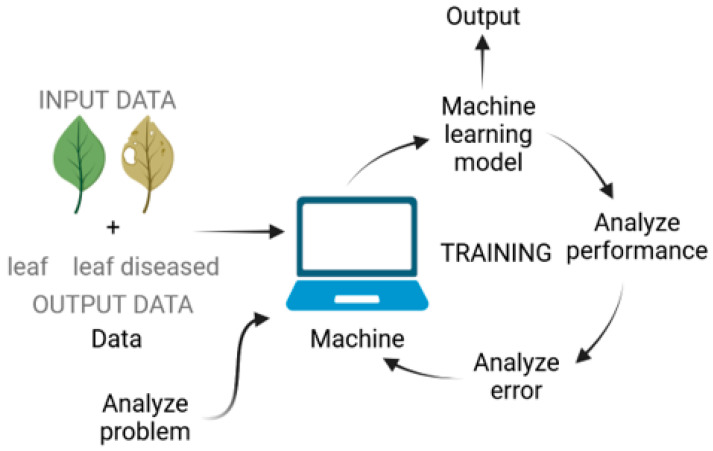
Machine learning approach to solving an object detection problem.

**Figure 3:**
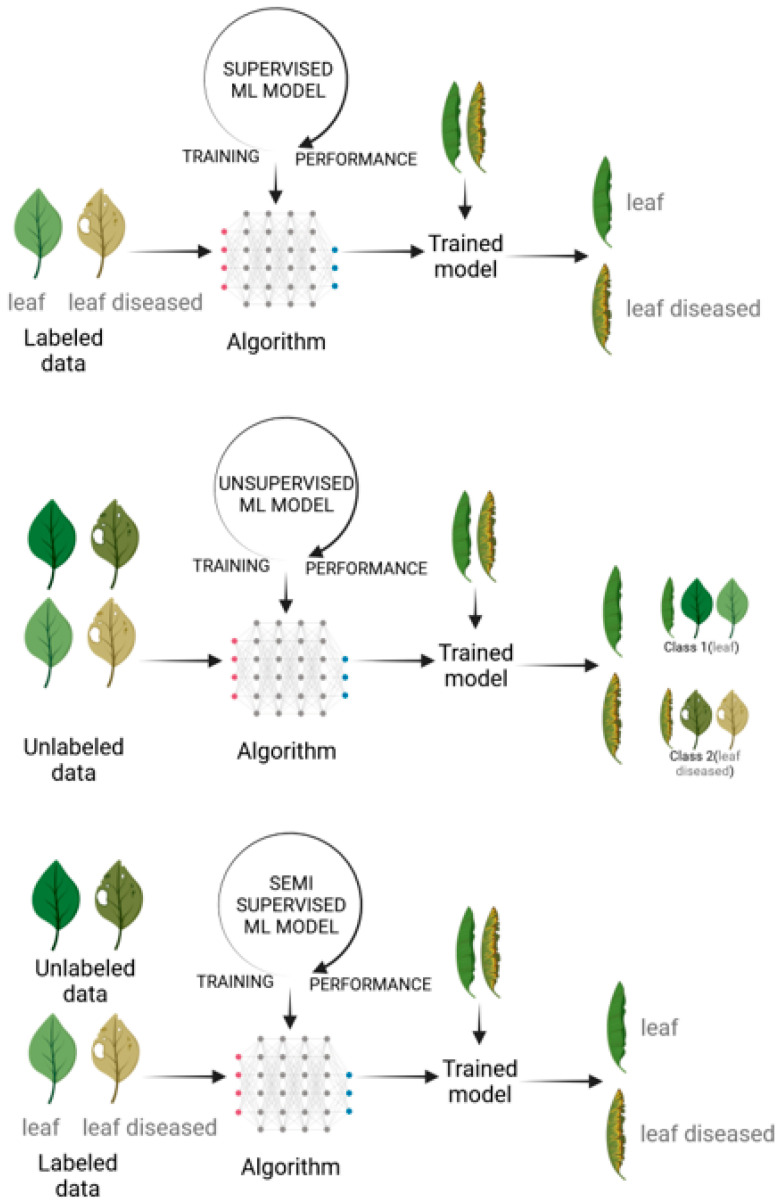
Machine learning approaches: supervised, unsupervised, and semi-supervised.

Machine learning is a subfield of AI that focuses on the development of algorithms and statistical models that enable computers to perform tasks without explicit instructions, but rather through patterns and inference. In other words, it’s a type of AI that allows a system to learn from data [136]. ML techniques differ from those of classical programming, which take input data and create a code to produce output data, and instead provide both inputs and outputs to generate algorithms (Figure 2). There are four main types of machine learning (Figure 3):

- Supervised learning: Involves learning a function that maps an input to an output based on example input–output pairs. It infers a function from labeled training data consisting of a set of training examples [137];
- Unsupervised learning: A type of machine learning that looks for previously undetected patterns in a data set with no pre-existing labels and with a minimum of human supervision [138];
- Semi-supervised learning: Combines supervised and unsupervised learning techniques;
- Reinforcement learning: An area of machine learning concerned with how software agents ought to take actions in an environment in order to maximize some notion of cumulative reward [139].

AI and ML have been increasingly used in horticultural research due to their ability to deal with complex data, extract patterns, and make predictions. They have found applications in a variety of areas, including plant phenotyping, disease detection, yield prediction, and stress identification, among others. In combination with multi-omics data, AI and machine learning can offer profound insights into plant biology, thus revolutionizing horticultural research and practice [28,140].

### 4.2. Importance of AI and Machine Learning in Data Analysis

The advent of AI and machine learning (ML) has significantly transformed the methods of analyzing data, particularly in the context of big data that is characteristic of many fields, including horticulture. Here, we outline the importance of AI and ML in data analysis:

- Handling high-dimensional data: One of the major challenges in modern horticulture research is dealing with high-dimensional data, often generated by high-throughput phenotyping and multi-omics technologies. AI and ML algorithms are particularly well-suited to handle such data, as they can process vast amounts of information efficiently, uncovering complex patterns and relationships that would be otherwise difficult to discern [141,142].
- Pattern recognition and feature extraction: ML algorithms excel at recognizing patterns within data. This is especially useful when dealing with complex biological data, where patterns may not be immediately obvious. ML can also be used for feature extraction, identifying the most informative variables within a dataset, which can greatly simplify data analysis and improve the accuracy of predictive models [98,99,100].
- Predictive modeling: AI and ML are powerful tools for predictive modeling. By learning from existing data, these algorithms can make accurate predictions about unseen data. This is especially important in horticulture, where predictive models can be used for various purposes, such as forecasting yield, predicting disease, or estimating the impact of environmental changes on plant growth and development [143,144,145,146,147].
- Dealing with noisy data: Real-world data often contains noise, which can complicate analysis and lead to erroneous conclusions. ML algorithms can effectively handle noisy data, extracting meaningful patterns while minimizing the impact of noise. This is particularly important in horticulture research, where data collected from field experiments can be influenced by a range of uncontrollable factors [148,149].
- Automating data analysis: AI and ML can automate many aspects of data analysis, making the process more efficient and less prone to human error. This can be especially beneficial when dealing with large datasets, where manual analysis would be time-consuming and impractical [150].
- Uncovering complex interactions: Biological data is often characterized by complex interactions and non-linear relationships. AI and ML algorithms, especially those based on deep learning, can model these complex interactions, providing a more accurate and holistic representation of biological systems [151,152].
- Integrating diverse data types: AI and ML provide a framework for integrating diverse types of data, such as genomic, transcriptomic, proteomic, metabolomic, and phenotypic data. This can facilitate a more comprehensive analysis and enable the extraction of more meaningful insights from the data [153,154].

The above techniques play a crucial role in modern data analysis, providing the tools necessary to extract meaningful insights from complex and high-dimensional data. As such, they have become an integral part of horticultural research, offering the potential to accelerate discoveries and improve our understanding of plant biology.

### 4.3. Potential of AI and Machine Learning in Horticulture Research

Artificial intelligence (AI) and machine learning (ML) have shown immense potential to transform horticulture research (Table 3). Here, we discuss some of the potential applications and implications of AI and ML in this field:

- High-throughput phenotyping: AI and ML are particularly promising for high-throughput phenotyping, helping to accurately analyze large volumes of data collected through imaging and sensor-based technologies. Automated image analysis, enabled by ML, can identify and quantify plant traits from these images, facilitating more precise and objective phenotypic measurements [155,156];
- Disease detection and diagnosis: AI and ML can aid in early disease detection and diagnosis by identifying patterns and anomalies in plant images or sensor data. This could help in monitoring plant health, predicting disease, and informing targeted interventions, thus minimizing losses due to diseases [157,158];
- Stress identification and quantification: ML models can help identify and quantify various biotic and abiotic stress factors, such as pests, diseases, drought, or nutrient deficiency, based on plant images, sensor data, or multi-omics data. This can contribute to a better understanding of plant responses to stress and the development of more resilient plant varieties [159,160];
- Yield prediction: AI and ML models can predict crop yields based on variables such as weather data, soil properties, and plant phenotypic data. Accurate yield prediction can assist in strategic decision-making and planning for growers and agricultural stakeholders [161,162];
- Genomic selection and breeding: AI and ML can assist in genomic selection and breeding by identifying genomic markers associated with desirable traits. This can accelerate the breeding process, enabling the development of improved plant varieties in shorter time frames [163,164];
- Integration with multi-omics data: AI and ML can be used to integrate and analyze multi-omics data, uncovering complex interactions and regulatory mechanisms that underlie plant traits. This can lead to a more comprehensive understanding of plant biology, informing both basic research and practical applications [57,165];
- Environmental monitoring and crop management: ML models can analyze data from various environmental sensors to monitor crop environments in real time and inform precision agriculture practices. This can help optimize resource use and maximize crop productivity and quality [19,20,166].

In conclusion, AI and ML hold significant potential to revolutionize horticulture research, contributing to advancements in plant phenotyping, disease diagnosis, stress identification, yield prediction, genomic selection, and precision agriculture. As these technologies continue to evolve, they are likely to provide increasingly powerful tools for addressing the complex challenges of modern horticulture.

### 4.4. A Machine Learning-Based Approach Using Multi-Omics Data: Preliminary Case Study

Multi-omics datasets are large and complex datasets which are generated from high-throughput technologies. Many integrated approaches are being sought out to aid in their analysis and visualization. Machine learning has been extensively used to analyze and integrate different types of data due to the increased accessibility of high computing power. These integrative approaches are continuously evolving to provide accurate insights from the data that is received through experimentation on various biological systems. This chapter describes the steps required for the ML–multi-omics integration methods that are applied to biological datasets for their analysis. We present the recommended algorithms used for integration and data analysis for supervised or unsupervised ML models.

If the data can be concatenated at an early stage:

- Unsupervised ML: Check if the multi-omics dataset is overlapping. If there is a partial overlap, MOFA2 (multi-omics factor analysis) [167] can be used. If the overlap is complete, check if there is a large dataset after integration. If yes, moCluster [168] and iClusterBayes [169] can be used; if no, iCluster [170] can be used. Next, check if the dataset has different distribution; if yes, JIVE (Joint and Individual Variation Explained) [171] and the JBF (joint Bayes factor) [172] can be used; if the dataset has similar distribution, NMF (non-negative matrix factorization) random forests (sklearn.decomposition.NMF) can be used.
- Supervised ML: Check if a large dataset is produced after integration. If yes, either ensemble methods such as the LASSO (Least Absolute Shrinkage and Selection Operator) [173] can be used. If we obtain a reduced dataset, it can be further solved using tools such as decision trees, the Naive Bayes model, SVMs (support vector machines), KNNs (k-nearest machines) [174], K-Star [175], boosted regression trees [176], SVR (support vector regression), ANNs (artificial neural networks), and DNNs (deep neural networks).

If the data can be concatenated at a later stage:

- Unsupervised ML: Tools such as FCA (formal concept analysis) consensus clustering [177], BCC (Bayesian consensus clustering) [178], and SNF (similarity network fusion) [179] can be used;
- Supervised ML: Tools such as hierarchical classifiers [180], ensemble-based classifiers (XGBoost and KNN), and autoencoder-based classifiers can be used.

If the dataset can be integrated as a transformation:

- Unsupervised: Check if the multi-omics datasets are overlapping. If the overlap is partial, NEMO (neighborhood-based multi-omics clustering) [181] can be used. If overlap is complete, Meta-SVM [182] can be used.
- Supervised: If it is a kernel-based transformation, tools such as SDP-SVM (semi-definite programming) [183], the RVM (Relevance Vector Machine) [184], and the AdaBoost RVM can be used. If it is a graph-based transformation, tools such as SSL (semi-supervised learning) [185], graph sharpening [186], and Bayesian networks, can be used.

Most ML workflows can be implemented on a standard Unix workstation in standard configuration. It can also be equipped with a graphics processing unit (GPU) to train ML models. The exact specifications of the machine would vary depending on the size of the dataset and model architecture. In addition to a CUDA-capable GPU and its suitable drivers, CUDA (https://developer.nvidia.com/cuda-toolkit; accessed on 14 September 2023) is an underlying parallel computing platform, which must be separately installed for training ML models. Additionally, multiple ML frameworks are available with active development and extensive community support, and are implemented in the Python programming language:

- Scikit-Learn: It is designed to work with Python’s NumPy and SciPy numerical and scientific libraries, and it includes support vector machines, random forests, gradient boosting, k-means, and DBSCAN, among other classification, regression, and clustering algorithms. To include Scikit-learn, import sklearn:

sklearn.cluster # All inbuilt clustering algorithms and functions are heresklearn.datasets # All inbuilt datasets are heresklearn.linear_model # All inbuilt linear models and functions are heresklearn.naive_bayes # To use the Naive Bayes modelsklearn.neighbors # To use the nearest neighbors modelsklearn.neural_network # To use neural network modelssklearn.svm # To use the support vector machine modelsklearn.tree # To use the decision tree modelsklearn.preprocessing # To use preprocessing and normalization techniquessklearn.ensemble # To use ensemble methods

- TensorFlow. It is designed to operate with tf.Tensor objects, which are multidimensional arrays or tensors, and makes ML faster and easier by utilizing Python for numerical calculation and data flow. To include TensorFlow, import tensorflow as tf:

tf.transpose(data) # Transpose given data elementstf.concat([data_1, data_2, data_3], axis = value)# Concatenate data elementstf.Variable([0.0, 0.0, 0.0]) # To store modelstf.keras # To bring the Keras functionalitiestf.examples.tutorials.mnist.input_data # To use the MNIST dataset

- Pytorch: It is production ready, with cloud support, a robust ecosystem, and dispersed training. To include Pytorch, import torch:

torch.Tensor([value]) # Define a tensortorch.randn(value_1, value_2. . .) # Define a matrix with random valuestorch.autograd # For automatic differentiationtorch.optim # Implement optimization algorithmstorch.nn # Neural network layer (sequential, linear, etc.)

It is generally recommended that all the required packages be installed in a virtual environment. This can be easily managed by any environment manager, such as Conda (https://docs.conda.io/en/latest/; accessed on 14 September 2023).

## 5. Current Applications of Multi-Omics and AI in Plant Phenotyping

### 5.1. Detailed Review of Existing Studies Employing These Techniques

In recent years, the integration of multi-omics data and AI/ML has gained momentum in plant phenotyping. Many studies have successfully employed these techniques to understand plant biology more comprehensively, enhance predictive modeling, and improve breeding strategies. Below, we review some of these key studies:

- Genomic selection and phenotypic prediction: Several studies have employed AI and ML techniques alongside genomics data for genomic selection and prediction of complex phenotypic traits. Montesinos-Lopez et al. (2018) [187] developed a deep learning algorithm for genomic-enabled prediction of complex traits in maize, wheat, and other crops. Their method significantly outperformed traditional genomic selection methods, demonstrating the power of ML in this context.
- High-throughput phenotyping: High-throughput phenotyping platforms generate vast amounts of data that can be analyzed using ML algorithms. Pound et al. (2017) [188] developed an ML-based root phenotyping system called “Deep Root”. This system uses convolutional neural networks (CNNs) to analyze images from X-ray computed tomography scans of plant roots, accurately quantifying root architecture traits.
- Disease detection: AI and ML, coupled with image analysis, have shown great potential in early detection and diagnosis of plant diseases. Barbedo (2018) [189] successfully employed deep learning models to identify plant diseases based on leaf images. This approach allows for the early detection of diseases, facilitating rapid and targeted responses to mitigate damage.
- Integration of multi-omics data: The integration of multi-omics data using AI and ML is an emerging area of research. Argueso et al. (2019) [190] utilized AI and ML to integrate genomic, transcriptomic, and epigenomic data in *Arabidopsis thaliana*. Their integrative approach revealed complex relationships between these different types of data and provided insights into the mechanisms underlying plant stress responses.
- Stress identification: AI and ML have also been used for the identification and quantification of plant stress. Singh et al. (2018) [191] applied ML algorithms to hyperspectral images of plants for the identification and classification of various biotic and abiotic stress conditions.

These studies collectively demonstrate the potential of integrating multi-omics data and AI in plant phenotyping. As our understanding of these tools deepens and technology continues to advance, we anticipate that their application will become increasingly commonplace and powerful, driving forward our understanding of plant biology and improving horticultural practices.

### 5.2. Success Stories and Limitations Encountered

While the integration of multi-omics data and AI/ML techniques in plant phenotyping has shown promising results, it has also encountered several limitations and challenges. In this section, we will present some of the success stories that have marked this field, such as:

- Predicting yield and quality traits: A significant success story involves using AI and ML for predicting yield and quality traits in crops. Machine learning models trained on genomic and phenotypic data have been successful in predicting complex traits in several crops, enhancing selective breeding programs. For example, a study by Zhou et al. (2021) [192] used AI models to accurately predict rice yield and quality traits, enabling faster and more precise selection in rice breeding programs;
- Disease identification and prediction: AI and ML have been successfully used for early disease detection and prediction in plants. Ferentinos (2018) [193] developed a deep learning model that accurately identified and classified plant diseases based on leaf images. This model facilitated early intervention, minimizing crop loss due to diseases.

Despite these successes, several limitations and challenges have been encountered in the integration of multi-omics data and AI/ML in plant phenotyping, such as:

- Data quantity and quality: A major challenge in the application of AI/ML techniques in plant phenotyping is the requirement for large quantities of high-quality data. The predictive performance of AI and ML models generally improves with larger training datasets. However, collecting large quantities of high-quality phenotypic and multi-omics data can be time-consuming and costly [194,195];
- Data integration: Integrating data from different omics layers is a complex task due to the differences in data types, scales, and structures. Furthermore, the biological interpretation of integrated multi-omics data can be challenging due to the complex and often non-linear relationships between different biological layers [166,196];
- Interpretability model: While AI and ML models can make accurate predictions, they are often seen as “black boxes” due to their complexity, making it difficult to interpret their predictions. This lack of interpretability can be a significant limitation, particularly in a scientific context where understanding the underlying biological mechanisms is crucial [197,198];
- Overfitting: AI and ML models, particularly more complex models, such as deep learning models, can be prone to overfitting, where they perform well on the training data but poorly on unseen data. This can limit the generalizability and predictive accuracy of these models [199,200].

While there have been notable success stories in the application of multi-omics data and AI/ML in plant phenotyping, several limitations and challenges need to be addressed to fully realize their potential. Continued research and development in these areas, along with the refinement of data collection and analysis techniques, are crucial for the future advancement of this field.

## 6. Integrated Multi-Omics and AI Framework

### 6.1. Description of the Proposed Framework

The proposed framework aims to integrate multi-omics data and artificial intelligence (AI)/machine learning (ML) techniques in order to gain deeper insights into plant phenotypes and to enhance predictive modeling capabilities in horticulture research. Here, we describe the key components and steps involved in this integrated framework:

1. Data collection: The framework begins with comprehensive data collection, encompassing multiple ‘omics’ layers—genomics, transcriptomics, proteomics, and metabolomics. Simultaneously, phenotypic data is collected using high-throughput phenotyping techniques. This may involve, for example, imaging technologies, environmental sensors, or manual trait measurements [201,202];
2. Data pre-processing and normalization: The collected data is pre-processed and normalized to ensure comparability and to minimize technical biases. This step may involve quality control, normalization, feature extraction, and other data transformation procedures [48,203];
3. Data integration: After pre-processing, data from different ‘omics’ layers is integrated. This integration can be done at various levels, for example, at the level of features (genes, transcripts, proteins, metabolites), samples, or phenotypes. Various data integration techniques, such as multivariate statistical methods, data fusion techniques, or network-based methods, can be used depending on the specific research question and data characteristics [201,204,205];
4. Machine learning modeling: Once the data is integrated, ML algorithms are employed to build predictive models or to extract meaningful patterns from the data. This may involve supervised learning methods for prediction tasks, unsupervised learning methods for data exploration, or reinforcement learning methods for decision-making tasks [201,206,207];
5. Model evaluation and interpretation: After the ML models are built, they are evaluated using suitable metrics and validation strategies. The interpretation of model results is also a crucial step, allowing for biological insights to be derived from the model’s predictions or patterns [208,209];
6. Application to horticulture research and practice: The final step involves applying the insights derived from the integrated multi-omics and AI/ML framework to horticulture research and practice. This could involve, for example, informing breeding strategies, enhancing disease detection and intervention methods, improving resource management, or predicting crop yields and quality [210,211].

### 6.2. How AI and ML Can Help in Integrating and Analyzing of Multi-Omics Data

Artificial intelligence (AI) and machine learning (ML) technologies offer transformative potential for the integration and analysis of multi-omics data. Below are several ways these technologies can facilitate this process:

1. Data integration: One of the major challenges in multi-omics research is the integration of diverse types of data, ranging from genomics to metabolomics. These data types often differ significantly in their structure, complexity, and size, making their integration a non-trivial task. AI and ML algorithms, such as matrix factorization, deep learning, and network-based methods, can be used to integrate these heterogeneous data types in a coherent way, enabling a more comprehensive view of biological systems [212,213,214];
2. Feature selection and extraction: AI and ML methods can help identify the most relevant features across different ‘omics’ layers. Techniques such as the LASSO, ridge regression, random forests, or deep learning can be employed to perform feature selection or extraction, helping to reduce dimensionality and to identify key genes, proteins, metabolites, or other features that are predictive of the phenotype of interest [215,216,217];
3. Pattern recognition: AI and ML excel in recognizing complex patterns in large and high-dimensional data, a task that is common in multi-omics research. Unsupervised learning methods, such as clustering, principal component analysis (PCA), or deep learning-based methods, can be used to detect patterns, correlations, or latent structures in multi-omics data, providing insights into the underlying biological mechanisms [134,218,219];
4. Predictive modeling: AI and ML techniques are powerful tools for building predictive models based on multi-omics data. Given the high-dimensional nature of multi-omics data, these techniques can be particularly useful for this task. For example, support vector machines, neural networks, or gradient boosting models can be used to predict phenotypes based on multi-omics data [146,220,221];
5. Network construction and analysis: AI and ML can also assist in the construction and analysis of biological networks based on multi-omics data. For instance, network-based methods can be used to infer gene regulatory networks, protein–protein interaction networks, or metabolic networks. These networks can provide valuable insights into the interactions and regulatory relationships between different biological entities [222,223,224].

In conclusion, AI and ML provide valuable tools for the integration and analysis of multi-omics data. By enabling data integration, feature selection, pattern recognition, predictive modeling, and network analysis, these technologies can greatly enhance our ability to understand and interpret multi-omics data, thereby contributing to advances in horticultural research.

### 6.3. Expected Benefits of the Proposed Framework

The integrated multi-omics and AI/ML framework offers significant benefits and is poised to significantly advance our proposed understanding and practices in horticultural research. Here are some of the anticipated benefits:

1. Enhanced understanding of plant biology: The framework’s ability to incorporate multi-omics data will allow for a more comprehensive understanding of plant biology, spanning from genes to metabolites. This in-depth view can reveal new insights into the complex mechanisms that govern plant growth, development, and responses to environmental conditions [130,225];
2. Improved predictive modeling: By leveraging the power of AI and ML, the proposed framework will enhance our capacity for predictive modeling. These advanced algorithms can manage the complexity and high dimensionality of multi-omics data, enabling more accurate predictions of plant traits and behaviors [226,227];
3. Accelerated breeding programs: The integration of multi-omics data and AI can expedite plant breeding programs. By accurately predicting desirable traits, breeders can make more informed selections earlier in the breeding cycle, thus reducing the time and resources required for breeding new varieties [228,229];
4. Optimized resource management: By predicting plant responses to different environmental conditions and management practices, the framework can guide decisions about resource allocation. This can lead to more sustainable and efficient use of resources such as water, fertilizers, and energy [230,231];
5. Enhanced disease diagnosis and intervention: The proposed framework can also improve disease detection and intervention strategies. AI and ML models can be trained to recognize early signs of disease based on multi-omics data, enabling early and targeted interventions that minimize crop damage [232];
6. Facilitating personalized horticulture: In the long term, the proposed framework could contribute to the development of ‘personalized horticulture’, where management strategies are tailored to the specific genetic makeup and environmental conditions of each plant or crop. This could lead to significant improvements in crop productivity, quality, and sustainability [233,234].

## 7. Challenges and Future Perspectives

### 7.1. Technical and Non-Technical Challenges in Implementing the Framework

The integrated multi-omics and AI/ML framework holds significant potential for advancing horticultural research and practice. However, its implementation also poses several technical and non-technical challenges that must be acknowledged and addressed (Table 4).

1. Technical Challenges:
  - Data acquisition and quality control: Collecting comprehensive multi-omics data is a complex and time-consuming task that requires specialized techniques and equipment. Ensuring the quality and consistency of this data across different ‘omics’ layers and samples is also a significant challenge [235,236];
  - Data integration: Integrating data from different ‘omics’ layers can be complex due to the differences in data types, scales, and structures. This task requires sophisticated methods and a deep understanding of both the data and the biological systems being studied [237,238];
  - Algorithm selection and implementation: Choosing and implementing the appropriate AI and ML algorithms for a given task can be challenging, particularly given the rapid pace of advancement in these fields. The chosen algorithms must be carefully validated and their assumptions and limitations understood [239,240];
  - Model interpretability: AI and ML models, particularly complex models, such as neural networks, can be difficult to interpret. This ‘black box’ nature can be a significant challenge in a scientific context where understanding the underlying mechanisms is crucial [241,242].
2. Non-Technical Challenges:
  - Ethical and legal considerations: The use of AI and ML in horticulture, like in many other fields, raises several ethical and legal considerations. These include issues related to data privacy and ownership, the transparency and fairness of AI/ML algorithms, and the potential impacts on labor markets [243,244];
  - Education and training: Implementing this framework requires a high level of expertise in various fields, including genomics, bioinformatics, AI and ML, and horticulture. Providing the necessary education and training can be a significant challenge [245,246];
  - Collaboration and communication: The interdisciplinary nature of this framework necessitates close collaboration and effective communication between experts in different fields. Overcoming disciplinary boundaries and fostering a collaborative culture can be a challenge [247,248].

Future research should focus on addressing these challenges and exploring potential solutions. By doing so, it will be possible to realize the full potential of the proposed framework and to drive significant advancements in horticultural research and practice.

### 7.2. Potential Solutions to These Challenges

Addressing the challenges associated with the implementation of the proposed multi-omics and AI/ML framework will require concerted efforts across several dimensions. Here are some potential solutions.

1. Solutions to Technical Challenges:
  - Standardization of data acquisition and quality control: Standardizing protocols for data acquisition and quality control can help ensure the comparability and consistency of multi-omics data. The development and adoption of universal standards and best practices across laboratories can be a key part of this process [249,250];
  - Development of sophisticated integration techniques: Continued research and development in the field of data integration can help overcome the challenges associated with integrating diverse ‘omics’ data. This includes not only statistical methods but also computational tools that can handle the complexity and size of multi-omics data [57,251];
  - Transparent and reproducible machine learning practices: Promoting transparency and reproducibility in AI and ML can help address the challenge of algorithm selection and implementation. This involves clearly documenting the choices made at each step of the ML process, making code and data available for others to reproduce results, and thoroughly validating and benchmarking algorithms [252,253];
  - Explainable AI: To tackle the ‘black box’ issue, efforts should be directed towards the development and application of explainable AI techniques. These methods aim to make the decision-making process of AI and ML models more transparent and interpretable [254,255].
2. Solutions to Non-Technical Challenges
  - Ethical and legal guidelines: To address the ethical and legal considerations associated with AI and ML, comprehensive guidelines and regulations should be developed and enforced. This should involve a wide range of stakeholders, including researchers, ethicists, legal experts, and policymakers [256,257];
  - Interdisciplinary education and training: The challenge of education and training can be addressed by promoting interdisciplinary education programs that provide a comprehensive understanding of both the biological and computational aspects of this field. This also includes continued professional development opportunities for researchers in this field [258,259];
  - Promoting collaboration and communication: Encouraging a culture of collaboration and communication can help overcome disciplinary boundaries. This can be facilitated by interdisciplinary conferences, workshops, and research projects, as well as tools and platforms that facilitate collaboration and data sharing [258,260].

By implementing these solutions, we can mitigate the challenges associated with the proposed framework, paving the way for the successful integration of multi-omics and AI/ML in horticultural research and practice.

## 8. Future Prospects of Integrating Multi-Omics and AI in Plant Phenotyping

The integration of multi-omics and AI in plant phenotyping promises a transformative future for horticultural research and practice. Here are some of the exciting prospects:

1. Precision horticulture: As we advance our ability to analyze and interpret complex multi-omics data using AI, precision horticulture becomes a promising reality. In this scenario, every decision, from planting to harvesting, can be tailored to the specific genetic makeup and environmental conditions of each plant, optimizing productivity, sustainability, and quality [55,261];
2. Predictive breeding: The combination of multi-omics and AI can vastly improve plant breeding processes. Breeders will be able to make informed decisions based on predictive models that take into account a comprehensive range of genetic and phenotypic data, significantly accelerating the breeding process and enhancing the resulting crop varieties [26,262];
3. Enhanced disease and stress response management: By integrating multi-omics and AI, we can achieve an unprecedented understanding of plant disease and stress responses. This could lead to the development of sophisticated early warning systems for disease and stress detection, as well as novel strategies for managing these challenges [26,261];
4. Sustainable crop management: With the combined power of multi-omics and AI, we can build robust models that account for the complex interactions between plants, soils, and climates. These models can inform sustainable management practices, leading to reductions in resource use and environmental impact [55,255];
5. Exploration of plant biodiversity: The proposed integrated framework allows for a deeper exploration of plant biodiversity. This can enhance our understanding of the rich variety of plant species and their adaptations, potentially uncovering new resources for breeding and conservation efforts [33,263];
6. Universal access to horticulture research: With the development of user-friendly AI tools and platforms for multi-omics data analysis, there’s potential for dissemination of horticulture research. This means that advanced plant phenotyping methods could become accessible to a broader range of researchers and practitioners, facilitating global advancements in this field [33,264].

## 9. Conclusions

The traditional methods of plant phenotyping, while foundational, have their limitations, particularly in their inability to capture the intricacy of plant biology. The emergence of genomics, transcriptomics, proteomics, and metabolomics, collectively known as multi-omics, enables a more comprehensive analysis. When coupled with the power of AI and machine learning, we have a potential toolset that can navigate the complexity and volume of multi-omics data effectively, providing meaningful interpretations and predictions that can revolutionize horticultural research and applications. However, the implementation of this integrated framework is not without challenges, both technical and non-technical. From data acquisition and integration to the application of suitable AI algorithms and their interpretation, there are still many technical obstacles around. In addition, ethical, legal, and educational considerations must be taken into account. We discussed potential solutions to these challenges, emphasizing the importance of standardization, the development of explainable AI techniques, the creation of comprehensive guidelines for ethical and legal considerations, interdisciplinary education, and fostering a culture of collaboration and communication. Looking ahead, the prospects of this integration are inspiring, encompassing precision horticulture, predictive breeding, improved disease and stress response management, sustainable crop management, exploration of plant biodiversity, and commercialization of horticulture research. 

## Figures and Tables

**Table 3:** AI and ML techniques used in horticulture.

AI/ML Technique	Description	Examples of Use in Horticulture
Supervised Learning	This is a type of machine learning where an AI is trained using labeled data. The AI then uses this training to predict the labels of new, unseen data [94].	Plant disease identification from images [122], yield prediction [116], fruit size and quality prediction [123], and weeding [124]. Estimation of microclimatic parameters in greenhouse cultivation [125].
Unsupervised Learning	This involves training an AI using data that has not been labeled. The AI identifies patterns and structures in the data itself [94].	Clustering of plant genotypes or phenotypes [126], identifying patterns in multi-omics data [127].
Reinforcement Learning	This is a type of machine learning where an AI learns to make decisions by performing actions and receiving feedback in the form of rewards or punishments [95].	Optimization of microclimatic conditions, such as lighting and irrigation and regulating the level of humidity in greenhouse crops [128,129].
Deep Learning	This is a subset of machine learning that uses artificial neural networks with many layers (hence the term “deep”). Deep learning can model complex, non-linear relationships [130].	Plant stress detection from hyperspectral imaging data [131], automated plant phenotyping from image data, disease prediction from multi-omics data [26].
Convolutional Neural Networks (CNNs)	These are deep learning models that are especially good at processing grid-like data, such as images [132].	Leaf disease detection from images, plant species identification from leaf images [133]. Detection of surface defects and early stages of fruit pathogen infection based on images [134].

**Table 4:** AI and ML techniques used in horticulture.

Type of Challenge	Description of Challenge	Potential Solutions
Technical	Managing the volume and complexity of multi-omics data [210].	Using advanced computational infrastructure, application of efficient data compression, normalization, and storage techniques [212].
Technical	Developing robust and transparent AI/ML models for complex biological data [214]	Application of interpretable machine learning algorithms, use of proper validation techniques, collaboration between data scientists and biologists [209].
Non-Technical	Need for multidisciplinary expertise (biology, bioinformatics, data science) in a single project [219]	Formation of multidisciplinary teams, collaboration between research institutions and universities, training programs for researchers [218]
Non-Technical	Ethical, legal, and social implications of using AI and multi-omics data in horticulture [216]	Development and enforcement of ethical guidelines, legislation, informed consent processes for data use, public engagement, and education [217].

## Data Availability

Not applicable.
